# 

**Published:** 1906-09-22

**Authors:** 


CONTENTS.

**The Unliveable Misery** - - - - - - - - 433
**The Depreciation of Medicine—Its Causes and Remedies** - - - - - - - - - - 434
**Annotations** - - - - - - - - - - - 435
**Medical Opinion and Movement** - - - - - 436
**Hospital Clinics—**
Observations on the Declining Birth-rate - - - - - 437
**Practical Notes on Diagnosis and Treatment** - 441
**New Clinical Methods—**
The Methylene Blue Reaction of Russo in Enteric Fever - - 442
A Key to the Theory of Opsonins - - - - - - 443
**Progress in Medicine and Surgery—**
Notes on Liquid Articles of Dietary - - - - - - 444
**Special Analytical and Health Commission on Tinned and Preserved Food** - - - - - 445
**Remedies and their Uses** - - - - - - - 446
**Hospital Administration—**
New Workhouse Infirmary at Stepping Hill, Stockport - - 447
The Fitting and Furnishing of Wards and Operating Theatres 448
Bread-making in Country Districts - - - - - 449
**Editor's Letter-Box** - - - - - - - - 449

*NURSING SECTION.*

**Notes on News from the Nursing World—**
The Duchess of Connaught and the Motor Accident—Sisters and Orderlies—Poor-law Nurses and Registration—Masquerading in Nurses' Uniform—A Local Government Board Inspector on Male Nurses—The Position in Italy—A Canadian Matron and her Nurses—Improvements at Carnarvon—An Experiment that Failed—An Incident at an Inquest—Medical Officer and Superintendent Nurse - Appointments to the Military Nursing Service—Victorian Nurses and Finance—Lectures to Midwives—A Photograph for Nurses—Dispensary Training in Hospitals—Short Items - - - - - - - - 351-354
**The Nursing Outlook** - - - - - - - 355
**Abdominal Surgery** - - - - - - - - - 356
**The Nurses' Clinic** - - - - - - - - - 357
**Incidents in a Nurse's Life** - - - - - - - 358
**Nursing at the Waterford County Infirmary** - - 359
**Our Mortuary** - - - - - - - - - 359
**Native Nursing in Italy** - - - - - - - 360
**The Open-air Treatment for Diarrhœa and Vomiting** - - - - - - - - - 361
**Presentations** - - - - - - - - - 361
**Everybody's Opinion** - - - - - - - 361
**Midwifery Appointment** - - - - - - - 363
**Novelties for Nurses** - - - - - - - 363
**Appointments** - - - - - - - - - 363
**Notes and Queries** - - - - - - - - 364
**For Reading to the Sick** - - - - - - - 364

